# Immunoregulatory Effects of Everolimus on *In Vitro* Alloimmune Responses

**DOI:** 10.1371/journal.pone.0156535

**Published:** 2016-06-08

**Authors:** Josh Levitsky, Joshua Miller, Xuemei Huang, Lorenzo Gallon, Joseph R. Leventhal, James M. Mathew

**Affiliations:** 1 Division of Gastroenterology & Hepatology, Department of Medicine, Northwestern University Feinberg School of Medicine, Chicago, Illinois, United States of America; 2 Comprehensive Transplant Center, Department of Surgery, Northwestern University Feinberg School of Medicine, Chicago, Illinois, United States of America; 3 Division of Nephrology, Department of Medicine, Northwestern University Feinberg School of Medicine, Chicago, Illinois, United States of America; 4 Department of Microbiology-Immunology, Northwestern University Feinberg School of Medicine, Chicago, Illinois, United States of America; University of Toledo, UNITED STATES

## Abstract

Everolimus (EVL) is a novel mTOR-inhibitor similar to sirolimus (SRL) that is used in organ transplant recipients, often in combination with tacrolimus (TAC) or mycophenolate (MPA). The current study aims to determine its effects on regulatory T cells. Increasing concentrations of EVL, MPA and TAC alone or in combination were added to MLRs of healthy volunteers. Lymphoproliferation by ^3^H-TdR incorporation and the percentage of newly generated CD4^+^CD127^-^CD25^+^FOXP3^+^ (total Treg) and CD4^+^CD127^-^CD25^High^FOXP3^+^ (natural Treg) in CFSE labeled responder cells were assessed by flow cytometry. In comparison to medium controls, EVL and other agents dose-dependently inhibited ^3^H-TdR incorporation in HLA-2DR-matched and HLA-mismatched MLRs (n = 3–10). However, EVL significantly amplified newly generated total and natural Tregs in CFSE labeled responder cells (p<0.05) at all concentrations, while MPA and SRL did this only at sub-therapeutic concentrations and inhibited at therapeutic levels. In contrast, TAC inhibited newly generated Tregs at all concentrations. When tested in combination with TAC, EVL failed to reverse TAC inhibition of Treg generation. Combinations of EVL and low concentrations of MPA inhibited proliferation and amplified Treg generation in an additive manner when compared to medium controls or each drug tested alone (p<0.05). The relative tolerogenic effect from high to low was EVL > SRL> MPA > TAC. If the results from these *in vitro* studies are extrapolated to clinical transplantation, it would suggest EVL plus low concentrations of MPA may be the most tolerogenic combination.

## Introduction

Although immunosuppression (IS) is effective in preventing rejection after organ transplantation (Tx), the standard IS agents, calcineurin inhibitors (CNI) such as tacrolimus (TAC), often lead to chronic renal impairment and other long term adverse effects. The search for improved treatment algorithms includes CNI dose minimization or withdrawal in favor of non-CNI treatment, such as the mTOR-inhibitors sirolimus (SRL) or everolimus (EVL) early enough before CNI toxicity is irreversible[1–5]. However, SRL instead of CNI early after transplant can increase the risk of rejection and graft loss, and its use particularly in liver transplantation (LTx) is not recommended [3]. Everolimus, however, is approved for use in LTx in combination with low dose TAC, but not as monotherapy [4–6]. Thus strategies to promote immunoregulation and allow for CNI minimization or possibly full withdrawal are greatly needed in the transplant population.

As a result of the development of an immune monitoring assay, in which allospecific T-regulatory cells are generated (the Treg-MLR) [7], we have analyzed the immunoregulatory effects of several commonly used IS agents. This assay, an *ex vivo* equivalent to transplantation alloimmunity, could be used to detect differences in mechanisms so as to optimize IS clinically in transplantation. Our results have demonstrated favorable effects on Treg generation for agents such as SRL, alemtuzumab, and mycophenolate (MPA) and inhibitory effects of agents such as TAC and CTLA4-Ig [7–11]. Clinically, we have previously demonstrated increased peripheral and graft Tregs during conversion from TAC to SRL in LTx recipients [1]. Now that EVL is being used clinically in LTx, one major question is if it has the same or greater *in vitro* and *in vivo* immunoregulatory properties well known for other mTOR inhibitors such as SRL [1, 8, 12, 13]. If this were determined to be so, EVL might be used in clinical trials of CNI minimization or even full IS withdrawal especially in LTx. In the present report, we have studied the effects of EVL in inhibiting alloimmunity and enhancing Treg generation *ex vivo* using the Treg-MLR assay [7].

## Materials and Methods

### Human Subjects and HLA Typing

The research was conducted on human subjects with the approval of the Northwestern Institutional Review Board (Study # STU 00002452) and conducted according to the principles expressed in the Declaration of Helsinki and also after obtaining written consent. PBMC were obtained from healthy volunteers who were HLA-typed and selected as either HLA-2DR-matched or HLA-mismatched.

### Culture media and IS agent additives

As previously described [1, 8, 9], the culture media (NAB-CM) consisted of RPMI 1640 with 15% normal human AB serum, 2mM L-glutamine, 10mM Hepes and 1X pen/strep/glutamine solution (GIBCO-BRL, Gaithersburg, MD).

Initially everolimus was diluted in NAB-CM at indicated concentrations and added at the culture outset, compared to cultures in which the same volume of media (no drug) was added. In later experiments combinations of EVL + TAC and EVL + MPA at therapeutic and sub-therapeutic concentrations were tested.

### Assessment of Proliferation in mixed lymphocyte reaction (MLR)

1x10^5^ responding PBMC from healthy volunteer (A) were cultured with 1x10^5^ irradiated stimulator cells from HLA-2DR-matched (B) or HLA-mismatched (I) laboratory volunteers in 96-well U-bottom culture plates in NAB-CM in triplicate. To these cultures the IS agent(s) either singly or in combinations were added at indicated concentrations. On day 7, 1uCi ^3^H-TdR was added to each well and after 18 hours the cultures were harvested using a Tomtec cell harvester (Hamden, CT). Radioactive incorporation was measured using a Packard-Beta counter (Meriden, CT). The Stimulation Index (SI) was calculated using the formula:

$$\text{Average CPM in allostimulated experimental conditions }\left(\text{E}\right)=\;\frac{\left(E1+E2+E3\right)}{3}$$

$$\text{Average CPM in autostimulated control conditions }\left(\text{C}\right)=\frac{\left(C1+C2+C3\right)}{3}$$

$$\text{Stimulation Index }\left(\text{SI}\right)=\frac{E}{C}$$

Percentage of Medium Control was calculated using the formula:

$${\text{SI in experimental cultures with immunosuppressive drugs added }=\text{ SI}}_{\text{E}}$$

$${\text{SI in control cultures without immunosuppressive drugs added }=\text{ SI}}_{\text{C}}$$

$$\text{\% of Media Control }\left(\text{MC}\right)=\left(\frac{SI_{E}}{SI_{C}}\right)\times 100$$

### The Treg-MLR

As previously described [1, 8, 9], after Ficoll-Hypaque gradient centrifugation, PBMC were labeled with CFSE following the manufacturer’s instructions (labeling efficiency of >99%). 5x10^5^ CFSE labeled responding PBMC from healthy volunteer (A) were cultured with 5x10^5^ PKH26 labeled stimulator cells from HLA-2DR-matched (Bx) or HLA-mismatched (Ix; indifferent 3^rd^ party) laboratory volunteers in 48-well culture plates. On days 0 (baseline), 5, 7 and 9 flow cytometric analyses were performed for surface markers using CD4-ECD, CD25-PC7, CD127-PE (all from Beckman-Coulter, Miami, FL) and for intracellular FOXP3-PC5 (eBioscience, San Diego, CA) as previously described [7, 8]. Gated viable lymphocytes were further gated followed for CFSE bright and dim cells which were negative for both CD127-PE and PKH26 (thus gating out CD127^+^ responders and any residual stimulators). This was followed by gating for CD4^+^ cells. CFSE dilution in these CD4^+^ responders assessed the extent of proliferation, i.e., non-proliferating (CFSE high) or proliferating (CFSE low) cells. The expression of CD25 and FOXP3 were analyzed in the non-proliferating and proliferating populations. Data were calculated as percentage of CD127^-^CD4^+^ cells that were CD25^+^FOXP3^+^ (total Tregs) or CD25^High^FOXP3^+^ (natural Tregs; nTregs) [10].

### Statistical Methods

Paired Student T-tests and Wilcoxon signed rank tests for parametric and nonparametric calculations respectively were used. P values ≤0.05 were considered statistically significant and are plotted as dotted line in the Figures. All statistical analyses were performed using SAS 9.2 statistical software (SAS Inc., Cary, NC).

## Results

### Direct effect of Everolimus in inhibiting both lymphoproliferation and phenotypic Treg generation in MLR

**Fig 1:**
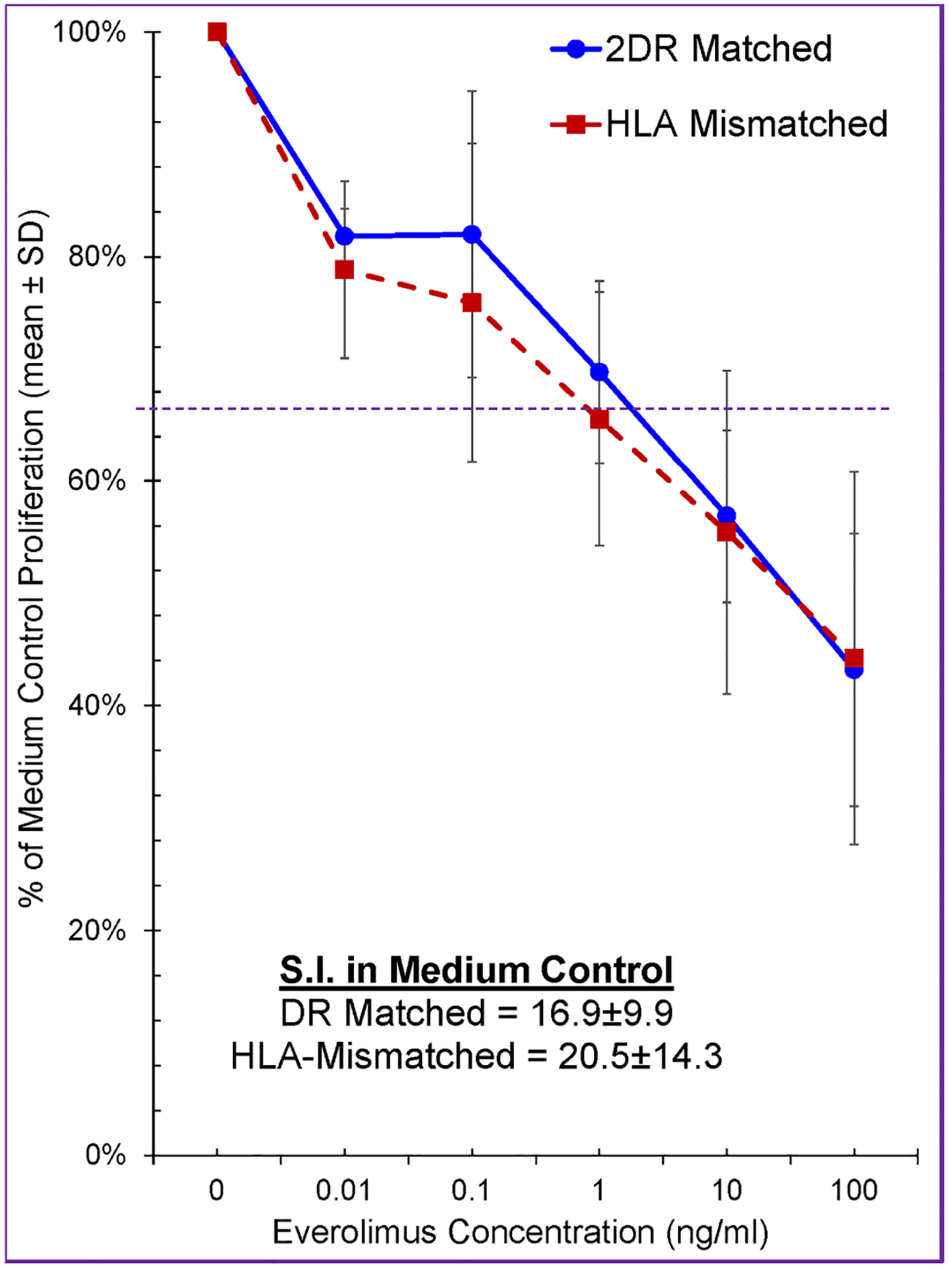
Effect of Everolimus on Lymphoproliferation in MLR (n = 7). 1x10^5^ responding PBMC from healthy volunteer A were cultured with 1x10^5^ irradiated stimulator cells from an HLA-2DR-matched volunteer (Bx) or HLA-mismatched laboratory third party indifferent volunteer (Ix), in the presence of the indicated concentrations of Everolimus. Standard ^3^H-TdR assays were performed on day 7 and the data were calculated as percentage of medium control (i.e. 100%). The dotted line indicates the level below which statistical significance at p≤0.05 was obtained. Note that Everolimus inhibited the MLRs in a dose dependent manner and that the degree of inhibition was equivalent against both DR-identical and HLA-mismatched stimulators.

Increasing concentrations of Everolimus (0 and from 0.01–100 ng/mL) were tested in MLRs using PBMC of healthy volunteers and a dose dependent inhibition of lymphoproliferation was observed (Fig 1). As expected the SI was higher in HLA-mismatched MLRs vs. HLA-2DR-matched MLRs (not shown). However, when the data were calculated in each experiment as % of medium control (100%), this distinction was cancelled out.

**Fig 2:**
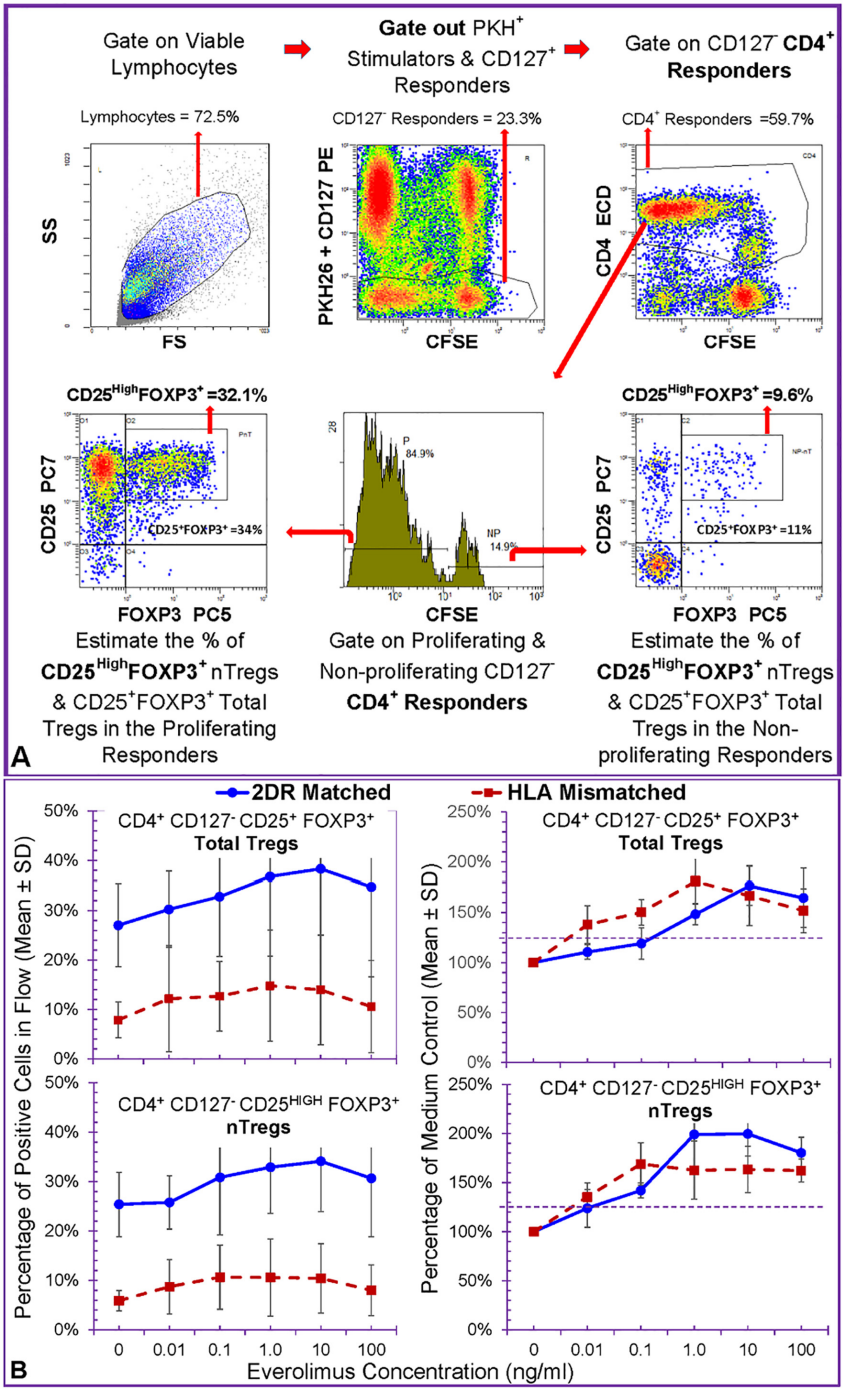
Effect of Everolimus on Treg Generation in MLR (n = 7). ***A*:** Parallel to the experiments described in Fig 1, 5x10^5^ CFSE-labeled PBMC responders (*A) were stimulated with 5x10^5^ PKH-26 labeled irradiated stimulators from HLA-2DR-matched (Bx) or completely HLA-mismatched (Ix) volunteers in the presence of indicated doses of Everolimus. Five color flow cytometric analysis was performed on days 0, 5, 7 and 9 (Day 0 = baseline; data from day 7 as the peak are shown here). Viable lymphocytes were gated followed by gating for CFSE bright and dim cells which were negative for both CD127-PE and PKH26 (thus gating out CD127^+^ responders and any residual stimulators). This was followed by gating for CD4^+^ cells. CFSE dilution in these CD4^+^ responders assessed the extent of proliferation, i.e., non-proliferating (CFSE high) or proliferating (CFSE low) cells. Then the percentage of CD4^+^CD127^-^CD25^+^FOXP3^+^ total Tregs or CD4^+^CD127^-^CD25^HIGH^FOXP3^+^ natural Tregs (nTregs) were estimated in the non-proliferating and proliferating responders. Shown here is a representative experiment to demonstrate the analysis scheme. **B:** On the left, the percentage of total Tregs (top) and nTregs (bottom) are depicted. On the right, the raw percentages for the Tregs obtained in each experiment are converted to percentage of medium controls (in which no Everolimus was added) to minimize the variations. Note that Everolimus enhanced the generation of both total Treg and nTregs in the proliferating fraction depicted here, without discernible effect on the non-proliferating fraction (not shown). This was more definitively seen in the clinically applicable drug concentrations tested (p<0.05).

Next we tested the effect of EVL on the generation of new regulatory T cells in the Treg-MLR with CFSE labeled responders and PKH26 labelled stimulators using the flow analysis scheme shown in Fig 2A. The percentage of CD4^+^CD127^-^CD25^+^FOXP3^+^ total Tregs and CD4^+^CD127^-^CD25^HIGH^FOXP3^+^ natural Tregs (nTregs) were estimated in the non-proliferating and proliferating responders. The percentages of total Tregs (top) and nTregs (bottom) depicted on the left in Fig 2B, showed that HLA-2DR-matched Treg-MLRs generated higher percentages of these cells. Since these raw Treg percentages have high variations from experiment to experiment, they were converted to percentage of medium controls (in which no EVL was added) for each experiment thus minimizing the variation (Fig 2B, right). Note that EVL enhanced the generation of both total Tregs and nTregs in the proliferating fraction depicted here, without discernible effect on the non-proliferating fraction (not shown). This was more definitively seen in the clinically applicable drug concentrations tested (p<0.05, indicated by the dotted line at 125%).

In subsequent experiments, only the HLA-2DR-matched MLRs and Treg-MLRs were performed as they generated more Tregs and as such partial matching might be more applicable in clinical transplantation in many centers. Similarly, since the kinetics of both total Tregs and nTregs were the same in all experiments (other than the total Tregs being slightly higher), only the data for the nTregs and as percentage medium control are shown in subsequent figures.

### Effects of Everolimus on MLRs in the presence of Mycophenolic Acid

**Fig 3:**
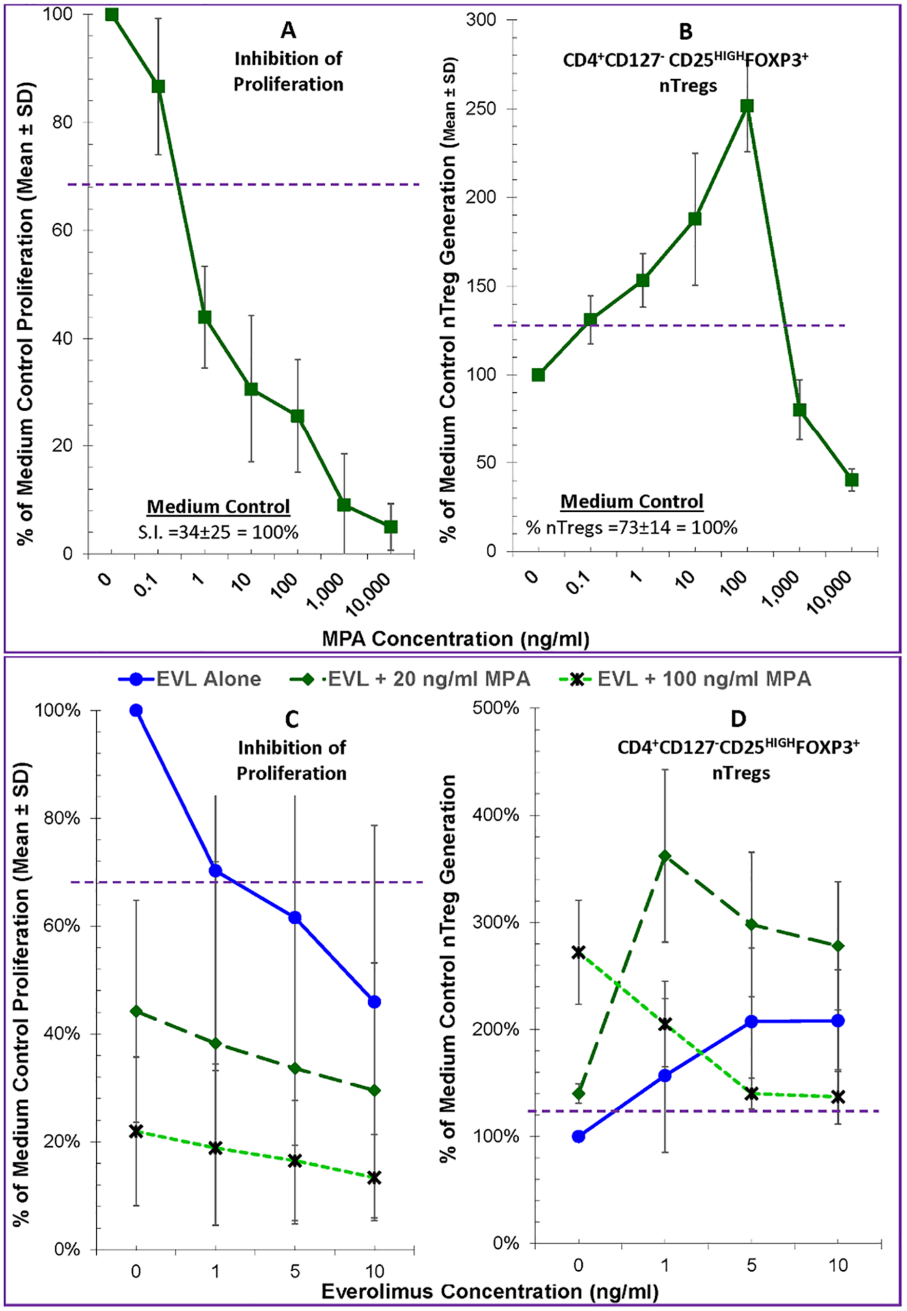
Effect of Everolimus plus Mycophenolic acid on allogeneic Lymphoproliferation and the generation of FOXP3^+^ Tregs in the Treg-MLR. **A.** Lymphoproliferation assays (left) and CFSE Treg-MLRs (right) as shown in Figs 1 and 2 respectively were performed, but in the presence of indicated concentrations of MPA (instead of EVL). MPA inhibited the proliferative responses in a dose dependent manner, and at subtherapeutic concentrations increased the generation of new Tregs in the responding cells (n = 4) (therapeutic is ~1-2ug/ml). **B.** EVL at indicated concentrations without or with 20 and 100 ng/ml MPA were tested in standard 7-day ^3^H-TdR assays lymphoproliferation assays (left) and in the CFSE Treg-MLRs (right). The data were calculated as percentage of medium control (i.e. 100%) to minimize variation among experiments (n = 4). (Left): Note that both EVL and MPA inhibited the MLRs in a dose dependent manner and that their combinations had an additive inhibitory effect on lymphoproliferation. Statistical significance was observed below the dashed line at 67% (p<0.05). (Right): MPA enhanced the generation of both subsets (comparing the 3 lines at 0 EVL) confirming results in Fig 3A, as was done by EVL (solid line). Combinations of EVL + MPA further amplified these Treg subsets (dashed lines) except at high concentration of MPA (dotted lines). Statistical significance was observed above the horizontal dashed line at 125% (p<0.05).

Since clinically EVL may be co-administered with mycophenolic acid (MPA) for maintenance immunosuppression, we tested combinations of these two drugs in the Treg-MLR. Initially, we tested the immune effects of a broad range of MPA, the *in vivo* active metabolite of mycophenolate mofetil (MMF). As shown in Fig 3A (left), MPA when tested alone inhibited the proliferative responses potently and in a dose dependent manner. However, it had a duality of effect on Tregs, at sub-therapeutic concentrations significantly increasing and at therapeutic concentrations (>1μg/ml) inhibiting new Treg generation in the responding cells (Fig 3A, right).

Based on these experiments, we tested two concentrations of MPA (20 and 100 ng/ml) in combination with various concentrations of EVL. As shown in Fig 3B, MPA by itself again inhibited lymphoproliferation in MLR in a dose dependent manner (comparing the 3 lines at 0 EVL). Similarly, EVL by itself inhibited proliferation (solid line in Fig 3B) confirming the results shown in Fig 1. The combinations of the two had additive or even synergistic inhibitory effect on allogeneic lymphoproliferation (dashed and dotted lines).

The effect of these two agents on Treg generation in MLR, however, was different (Fig 3B, right). As expected from the results shown Fig 3 (right), both 20ng/ml and 100ng/ml MPA amplified Treg generation (comparing the 3 lines at 0 EVL in Fig 3B, right). Likewise, EVL induced the generation of new Tregs in the proliferating responder cells (solid line in Fig 3B, right). When used in combination, lower concentration of EVL and MPA had additive enhancing effect (dashed line). However, at higher combinatorial concentrations, this Treg amplification was abrogated (dotted line), possibly secondary to the near total inhibition of proliferation (Fig 2B left, dotted line). These results would indicate that therapeutic clinical concentrations of EVL (3-8ng/ml) would amplify immune regulation; however, clinically applicable concentrations of MPA (1–4μg/ml) might abrogate this beneficial effect.

### Direct effects of combinations of Everolimus and Tacrolimus

**Fig 4:**
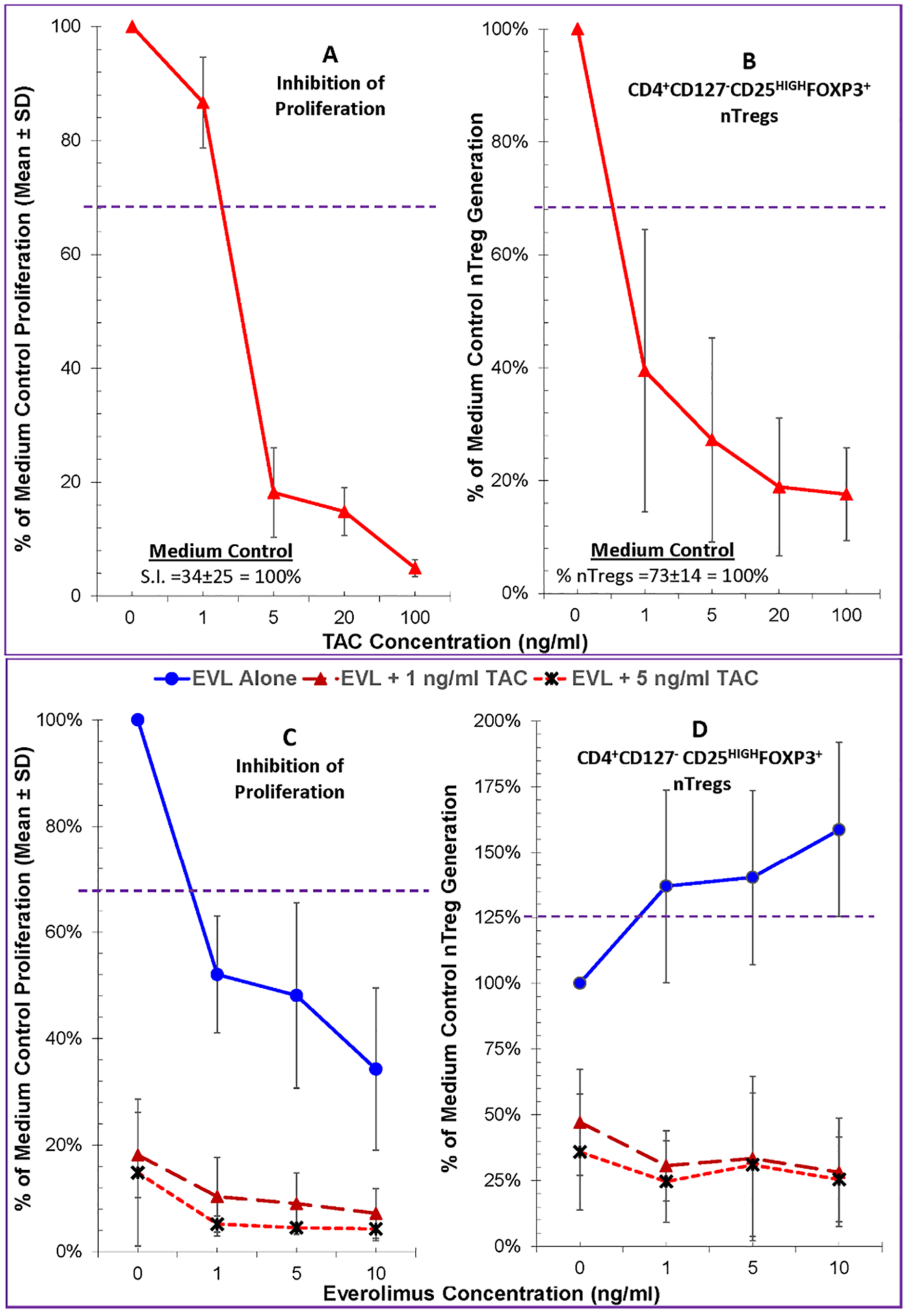
Effect of Everolimus plus Tacrolimus on allogeneic Lymphoproliferation and the generation of FOXP3^+^ Tregs in the Treg-MLR. **A.** Lymphoproliferation assays (left) and CFSE Treg-MLRs (right) as shown in Figs 1 and 2 respectively were performed, but in the presence of indicated concentrations of TAC (instead of EVL). TAC inhibited the proliferative responses and the generation of new Tregs in the responding cells (unlike other agents) in a dose dependent manner (n = 7). **B.** EVL at indicated concentrations without or with 1 and 5 ng/ml TAC were tested in standard 7-day ^3^H-TdR assays lymphoproliferation assays (left) and in the CFSE Treg-MLRs (right). As in other experiments, the data were calculated as percentage of medium control (i.e. 100%) (n = 4). (Left): Note that both EVL and TAC inhibited the MLRs in a dose dependent manner and that their combinations had an additive inhibitory effect on lymphoproliferation. Statistical significance was observed below the horizontal dashed line at 67% (p<0.05). (Right): TAC inhibited the generation of new Tregs (comparing the 3 lines at 0 EVL) confirming results in Fig 4A, and unlike by EVL (solid line). When combinations of EVL + TAC were tested, EVL failed to abrogate the inhibition mediated by TAC.

Clinically EVL is co-administered with Tacrolimus (TAC) for maintenance immunosuppression especially early (>1 month) after LTx. Therefore, we tested combinations of these two drugs in MLR and Treg-MLR. When tested at a broad range encompassing below, at and above therapeutic levels, TAC inhibited the proliferative responses potently and in a dose dependent manner (Fig 4A, left). Unlike EVL or MPA, however, TAC at any concentration tested inhibited new Treg generation in the responding cells (Fig 4A, right).

Next we tested a sub-therapeutic (1ng/ml) and therapeutic (5ng/ml) concentrations of TAC in combination with various concentrations of EVL. As shown in Fig 4B (left), TAC by itself again inhibited lymphoproliferation in MLR in a dose dependent manner (comparing the 3 lines at 0 EVL). Similarly, once again, EVL also by itself inhibited proliferation (solid line in Fig 4B, left). The combinations of the two had additive or even synergistic inhibitory effect on allogeneic lymphoproliferation (dashed and dotted lines).

The effect of these two agents on Treg generation in MLR, however, was drastically different (Fig 4B, right) from those seen with MPA. As expected both TAC at 1ng/ml and 5ng/ml MPA inhibited (comparing the 3 lines at 0 EVL), and EVL induced the generation of new Tregs in the proliferating responder cells (solid line). When used in combination, however, any of the concentrations of EVL tested, including those clinically relevant, failed to abrogate the inhibition of Treg generation mediated by TAC (dashed and dotted lines).

### The Comparative Effects of Everolimus vs. Sirolimus on Treg Generation

Everolimus is now the mTOR-inhibitor of choice in transplant recipients and is approved for use in both kidney and liver transplant populations. Sirolimus is however contraindicated in liver recipients due to an increased risk of adverse events; however, the vast majority of data on the immunoregulatory potential of mTOR-inhibitors has come from study of sirolimus, including our own work, with little data on EVL. Thus, with increased use of EVL in clinical practice and tolerance protocols, we were interested in determining if EVL had similar tolerogenic effects to SRL that could support such clinical applications.

**Fig 5:**
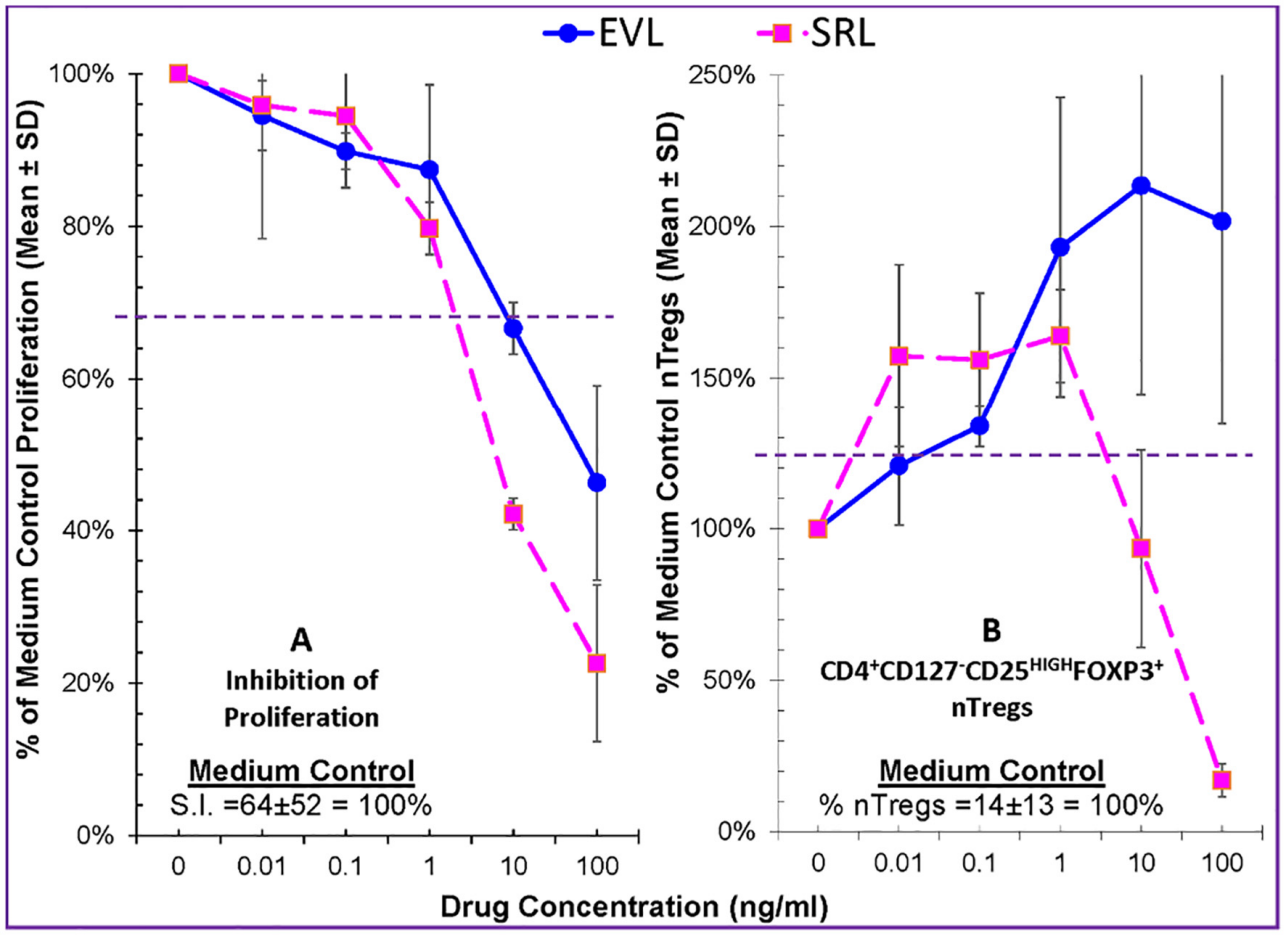
Comparison of Everolimus versus Sirolimus on allogeneic Lymphoproliferation and the generation of FOXP3^+^ Tregs in the Treg-MLR. Lymphoproliferation assays (left) and CFSE Treg-MLRs (right) as shown in Figs 1 and 2 respectively were performed, in the presence of indicated concentrations of EVL or SRL. As in other experiments, the data were calculated as percentage of medium control (i.e. 100%). (Left): Both EVL and SRL inhibited the proliferative responses in a dose dependent manner. Statistical significance was observed below the horizontal dashed line at 67% (p<0.05) (n = 4). (Right): Compared to no drugs, SRL augmented the generation of new Tregs at subtherapeutic concentrations; but inhibited their generation at therapeutic (5-10ng/ml) concentrations. However, EVL amplified the generation of new Tregs nearly at a dose-dependent manner, even to the supra-therapeutic concentration of 100ng/ml. The dotted line at 125% represents statistical significance at p<0.05 (n = 4).

We first compared EVL and SRL in inhibition of proliferation, and both similarly inhibited in dose-dependent fashion from subtherapeutic, therapeutic, and supratherapeutic concentrations (Fig 5—left panel). We then compared nTreg generation in Treg-MLR and both drugs expanded Tregs at sub-clinical concentrations (<1 ng/ml) (Fig 5—right panel). However at therapeutic and even higher concentrations (10–100 ng/ml), EVL continued to expand nTregs while SRL inhibited their generation. This suggests that EVL is a more potent and consistent immunoregulatory agent and less dependent on concentrations for these effects, which is useful in clinical practice where therapeutic blood levels can vary even at steady-state.

## Discussion

Everolimus (EVL) is a novel mTOR-inhibitor similar to sirolimus (SRL) that is used in organ transplant recipients, often in combination with other IS agents. Its main use is in kidney and liver transplant recipients early after transplant to protect against rejection and reduce the nephrotoxic effects of CNI therapy [2, 4, 5]. However, very little information is available on the immunoregulatory properties on EVL [14, 15] and if they are different or similar to those of SRL which are well established [1, 8, 12, 13, 15–17]. Therefore, we have utilized our Treg-MLR assay to robustly assess its *in vitro* effects singly or in combination with and compared to other agents [7].

Our *in vitro* analysis demonstrates that EVL possesses immunoregulatory properties typical of mTOR-inhibitors [1, 8, 12, 13], perhaps even greater than expected. As predicted, each of the IS agents studied (TAC, EVL, MPA, SRL) and clinically used in organ transplant recipients inhibited lymphoproliferation. With regard to Treg generation, however, EVL had the most profound impact at all clinical concentrations. As we have seen previously, drugs like MPA have *in vitro* immunoregulatory effects but mainly at sub-therapeutic doses [9]. This is confirmed in our current study. Additionally, these results showed that the combination of EVL plus MPA inhibited proliferation and amplified Treg generation in an additive manner (Fig 3B). Also, consistent with previous studies, TAC had profound Treg inhibitory effects (Fig 4), i.e., abrogated the positive mTOR-inhibitor effects, even of EVL [8, 18, 19].

Mechanistically, a key difference between CNI and mTOR-inhibitors is their effect on both Tregs and regulatory ‘immature’ dendritic cells (DCregs) important in the suppression of immune activation. CNIs inhibit IL-2 transcription and thus negatively affect Treg production [12, 16, 20]. In contrast, mTOR-inhibitors like SRL and presumably EVL, function at a later stage after T cell activation as inhibitors of interleukin-2 (IL-2) signaling, blocking proliferation of effector T cells, but still potentially promoting Tregs, DCregs and regulatory cytokines (i.e. TGF-β1) [13, 21]. Regulatory T cells may be more resistant to the immunosuppressive effects of mTOR- inhibitors as they inhibit post-activation signaling (i.e. PI3K/mTOR pathway) and have less effects on pathways (i.e. STAT5) involved in Treg generation [17, 22]. Finally, SRL can induce FOXP3 generation in antigen-specific T cells in the context of cytokines such as TGF-β [12, 22]. We have utilized a number of *in vitro* assays testing donor-specific Treg inhibition/recruitment (Treg-MLR in this study) and regulation of cytotoxic T cells (micro-cell mediated lympholysis) induced by IS therapies and in clinical tolerance trials [7, 8, 23]. We have found that SRL has allospecific immunoregulatory effects in the Treg-MLR [8]. In addition, we have demonstrated that direct TAC to SRL conversion in LTx recipients significantly increases systemic Tregs (blood, bone marrow, allograft) and DCregs, as well as proteogenomic signatures of immunoregulation [1, 24].

While the effects on Treg generation *in vivo* and *in vitro* are well established with SRL, prior studies have not robustly investigated whether EVL has similar effects in the allo-immune/organ transplant setting. This is clinically relevant given EVL, in contrast to SRL, is approved for use in both liver and kidney transplant recipients and seemingly has a more favorable side effect profile [2, 4]. In a study examining the potential for immunological tolerance by Treg vaccination in mice, Daniel *et al* found that EVL potently enhances Treg conversion by interfering with T-cell co-stimulation and activation [25]. Also, similar to our work, Game *et al* demonstrated that EVL, unlike CNIs, does not inhibit the suppressive effects of human Tregs *in vitro* [14]. Preliminary clinical studies have demonstrated an increase in Tregs following both EVL and SRL conversion from CNI therapy in organ recipients, correlating with suppression of activated CD4 cells [26, 27].

Interestingly, we have demonstrated inherent differences between the two mTOR-inhibitors on Treg generation across the various drug concentrations. Everolimus expanded Tregs at all concentrations, whereas SRL actually inhibited Treg generation at higher concentrations. This suggests that EVL is a more potent and consistent immunoregulatory agent, which is useful as an approved drug in clinical practice where therapeutic blood levels can vary even at steady-state. The exact mechanisms that explain Treg generation differences between EVL and SRL are not clear and need further examination. To date, there are very little data from the literature comparing the two mTOR-inhibitor effects on Tregs, let alone mechanistic studies. However, two recent papers shed light on potential mechanisms and the differences between the two mTOR-inhibitors that may explain our findings. Merino *et al* compared proliferation and intracellular cytokine production of different T cell subsets under the influence of calcineurin and mTOR-inhibitor therapies [28]. This demonstrated that across all concentrations, EVL led to continued high production of intracellular IL-2 in naïve T cells, where it was suppressed with increasing SRL concentrations, similar to CNI therapy. This greater IL-2 effect might explain why we saw continued Treg generation even at high EVL concentrations in our study, while IL-2 inhibition at high SRL concentrations could have led to lower Treg frequencies. In addition, in Merino’s study, EVL dose-dependently maintained naïve T cell phenotypes and inhibited central memory T cells more potently than SRL, which can both lead to greater Treg generation (e.g. naïve T cells have more plasticity to convert to Tregs under certain cytokine environments). Next, Sabbatini *et al* found a clear dose dependent relationship between Treg generation and EVL via the degree of mTOR inhibition [29]. Specifically, higher EVL concentrations were associated with lower T-cell receptor triggered S6 kinase phosphorylation in the mTOR activation pathway, which has not been seen with SRL. These data support both increased IL-2 production and inhibition of mTOR activation as the mechanism behind the more potent EVL Treg effects. That said, now that we have demonstrated differences in mTOR-inhibitors herein, we need to perform similar functional MLR assays comparing IL-2 and other pro-Treg cytokines, as well as mTOR pathway signaling, between the two mTOR-I agents—to confirm these hypotheses more rigorously.

The clinical relevance of utilizing immunoregulatory treatment in organ transplantation stems from an increasing interest to facilitate IS minimization or withdrawal. A number of IS withdrawal trials are now focusing on augmenting natural or induced Tregs to achieve tolerance, either by direct infusion of allo- or non-specific Tregs as therapy or conversion to more immunoregulatory IS therapy [30]. As mentioned previously, we recently demonstrated that liver recipients converted from TAC to SRL develop systemic signatures of immunoregulation [1]. Given these and our current *in vitro* results, we might expect similar or greater clinical Treg augmentation with EVL. While this needs confirmation, safe CNI to EVL conversion might represent an important clinical intervention toward full IS withdrawal, as direct CNI withdrawal in this context is often unsuccessful unless performed years after transplantation [31, 32]. In addition, the higher rejection rate seen with early post-LTx CNI to EVL conversion might be offset by adding MPA [6], supported by our findings that the combination of EVL and MPA is the most immunoregulatory. This might allow for the renal and other metabolic benefits of early CNI withdrawal, and an increased future potential of IS weaning. It should be noted that one of the potential limitations of our study is that any *in vitro* assay may not mimic the *in vivo* environment in transplant patients, given that drug levels may vary substantially *in vivo* compared to our controlled cultures. However, we believe our *in vitro* studies [7, 9, 33], with clinically relevant drug combinations, set the stage to determine if EVL is as immunoregulatory *in vivo* and, as such, can facilitate more favorable management strategies in such patient populations.

## Abbreviations

^3^H-TdR: Tritiated Thymidine
CFSE: Carboxyfluorescein succinimidyl ester
CPM: Counts per minute
EVL: Everolimus
FOXP3: Forkhead/winged helix transcription factor P3
HLA: Human leukocyte antigen
IS: Immunosuppression
MHC: Major Histocompatibility Complex
MLR: Mixed Lymphocyte Reaction
MPA: Mycophenolic Acid (in vivo active form of Mycophenolate)
PBMC: Peripheral blood mononuclear cells
SI: Stimulation Index
SRL: Sirolimus
TAC: Tacrolimus
Treg: T-regulatory cell
